# Longitudinal real world correlation study of blood pressure and novel features of cerebral magnetic resonance angiography by artificial intelligence analysis on elderly cognitive impairment

**DOI:** 10.3389/fnagi.2023.1121152

**Published:** 2023-02-03

**Authors:** Shasha Sun, Dongyue Liu, Yanfeng Zhou, Ge Yang, Long-Biao Cui, Xian Xu, Yuanhao Guo, Ting Sun, Jiacheng Jiang, Na Li, Yabin Wang, Sulei Li, Xinjiang Wang, Li Fan, Feng Cao

**Affiliations:** ^1^Department of Cardiology, Chinese PLA Medical School, The Second Medical Center and National Clinical Research Center for Geriatric Diseases, Chinese PLA General Hospital, Beijing, China; ^2^School of Artificial Intelligence, University of Chinese Academy of Sciences, Beijing, China; ^3^Laboratory of Computational Biology and Machine Intelligence, National Laboratory of Pattern Recognition, Institute of Automation, Chinese Academy of Sciences, Beijing, China; ^4^Department of Radiology, The Second Medical Center and National Clinical Research Center for Geriatric Diseases, Chinese PLA General Hospital, Beijing, China; ^5^Nankai University School of Medicine, Nankai University, Tianjin, China

**Keywords:** blood pressure, cognitive impairment, posterior circulatory artery, stenosis, aging

## Abstract

**Objective:**

This study aims to investigate novel clinical risk factors for cognitive impairment (CI) in elderly.

**Methods:**

A total of 3221 patients (259 patients with CI and 2,962 subjects without CI) were recruited into this nested case-control study who underwent cerebral magnetic resonance angiography (MRA) from 2007 to 2021. All of the clinical data with MRA imaging were recorded followed by standardization processing blindly. The maximum stenosis score of the posterior circulatory artery, including the basilar artery, and bilateral posterior cerebral artery (PCA), was calculated by the cerebral MRA automatic quantitative analysis method. Logistic regression (LR) analysis was used to evaluate the relationship between risk factors and CI. Four machine learning approaches, including LR, decision tree (DT), random forest (RF), and support vector machine (SVM), employing 5-fold cross-validation were used to establish CI predictive models.

**Results:**

After matching with age and gender, 208 CI patients and 208 control subjects were finalized the follow-up (3.46 ± 3.19 years) with mean age at 84.47 ± 6.50 years old. Pulse pressure (PP) in first tertile (<58 mmHg) (OR 0.588, 95% confidence interval (CI): 0.362–0.955) was associated with a decreased risk for CI, and ≥50% stenosis of the left PCA (OR 2.854, 95% CI: 1.387–5.872) was associated with an increased risk for CI after adjusting for body mass index, myocardial infarction, and stroke history. Based on the means of various blood pressure (BP) parameters, the performance of the LR, DT, RF and SVM models accurately predicted CI (AUC 0.740, 0.786, 0.762, and 0.753, respectively) after adding the stenosis score of posterior circulatory artery.

**Conclusion:**

Elderly with low pulse differential pressure may have lower risk for cognitive impairment. The hybrid model combined with the stenosis score of posterior circulatory artery, clinical indicators, and the means of various BP parameters can effectively predict the risk of CI in elderly individuals.

## 1. Introduction

It is reported that 1.6 billion people will be aged 65 years or older and half a billion people will reach 80 years or older by 2050 worldwide (He et al., 2016). Human life expectancy has increased nearly 2-fold in the past century (He et al., 2016). At present, 18.7% of the 1.44 billion Chinese people is aged more than 60 years (National Bureau of Statistics, 2021a,b), in which there is a dramatic increasing rate of mild cognitive impairment (MCI) and dementia (Jia et al., 2020; Pais et al., 2020). Early detection and precise intervention of neurodegenerative diseases are critical for improving life quality among elderly.

Previous studies have shown that hypertension is one of the most vital modifiable risk factors for cognitive impairment (CI) (Livingston et al., 2017). At present, the incidence of hypertension in patients with MCI is as high as 56.9% (Jia et al., 2020). But few studies have kinetic assessed the effect of blood pressure (BP) on the risk of MCI or dementia (Du et al., 2019). Abnormal BP is also one of the important causes of CI associated with a greater risk of cerebral artery stenosis (Maarten et al., 2020). Higher systolic blood pressure (SBP) and pulse pressure (PP) are associated with arterial stenosis in elderly individuals (Song et al., 2020). Some studies have reported that posterior cerebral arterial lesions are independent risk factors for dementia and MCI (Casado-Naranjo et al., 2016; Suri et al., 2018). Three dimensional time-of-flight magnetic resonance angiography (3D-TOF MRA) can clearly display the morphology of cerebral arteries to monitor vascular diseases. At present, there is a lack of sensitive and objective values of the cerebral circulatory artery stenosis for estimating CI by cerebral MRA. The present study was designed to analyze risk factors for CI by artificial intelligence (AI) analysis based on the BP parameters and cerebral magnetic resonance angiography (MRA) for the early prediction of CI in elderly individuals.

## 2. Materials and methods

### 2.1. Study population

A retrospective nested case-control study was designed to evaluate the association between risk factors and CI. A total of 3221 elderly patients (more than 65 years old) who underwent cerebral MRA scans in the Second Medical Center of the Chinese PLA General Hospital from 1 January 2007, to 30 April 2021 were included in this study.

**FIGURE 1:**
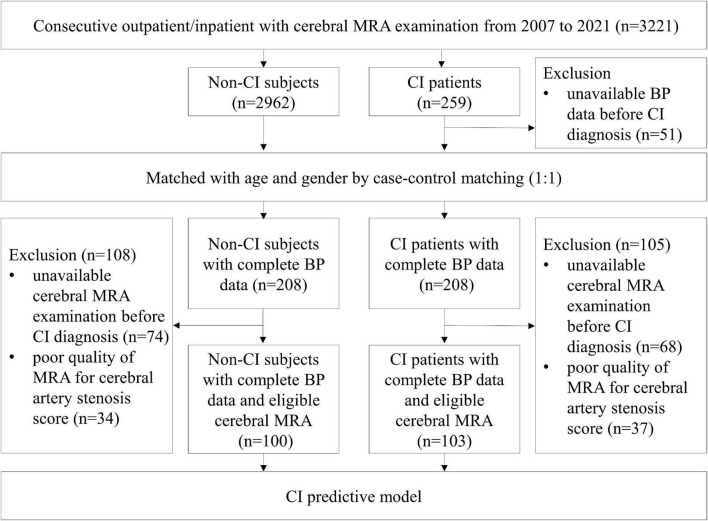
Flow chart of sample selection. Among 3,221 consecutive outpatient and inpatient who underwent cerebral MRA examinations, 208 CI patients and 208 age- and sex-matched non-CI subjects with complete BP data before onset of CI were applied to analyze the relationship between the means of various BP parameters and CI. In addition, 103 CI patients and 100 control subjects with eligible cerebral MRA performed before onset of CI were applied to analyze the relationship between posterior circulatory artery score and CI. BP, blood pressure; CI, cognitive impairment; MRA, magnetic resonance angiography.

Inclusion criteria: (i) Diagnosis of CI or without CI by neurologist in the medical records; (ii) BP data available before diagnosing CI or without CI; (iii) age 65 years or above, (iv) survival when the case patient was diagnosed with CI. Exclusion criteria: (i) missing information on BP; (ii) BP data unavailable before diagnosing CI; (iii) other diseases imparting a life expectancy of less than 6 months. A total of 208 CI patients with complete BP data who were diagnosed during the course of the study period were screened as the CI group. In addition, 208 age- and sex-matched non-CI subjects were randomly selected from the same cohort by case-control matching. For automated quantitative analysis of the posterior circulatory artery, 103 subjects with complete BP data and eligible cerebral MRA in the CI group, while 100 subjects in the control group were finished screening (Figure 1). All subjects were with eligible cerebral MRA imaging for the vascular auto-segmentation.

**FIGURE 2:**
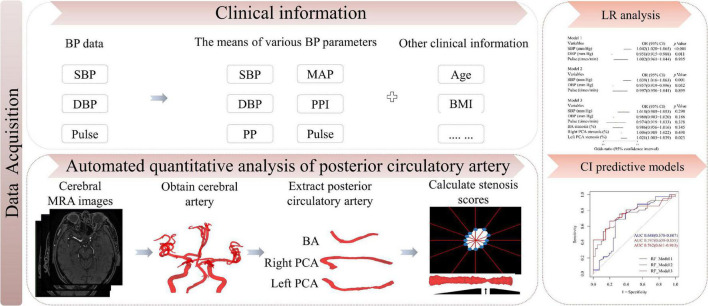
Study flow chart. BP, blood pressure; SBP, systolic blood pressure; DBP, diastolic blood pressure; MAP, mean arterial pressure; PP, pulse pressure; PPI, pulse pressure index; BMI, body mass index; LR, logistic regression; CI, cognitive impairment; MRA, magnetic resonance angiography.

The definition of CI (including MCI or dementia) was based on the ICD-10 of the WHO. Abnormal BP was diagnosed according to the Chinese hypertension guidelines (Chinese Hypertension League et al., 2019) and categorized into three tertiles. Diagnoses and assessments of MCI, dementia and abnormal BP were adjudicated by two senior neurologists. All examinations and information collection were completed blindly by two professional clinicians. This study protocol was approved by the ethics committee on human experimentation of the Chinese PLA General Hospital and all protocol were implemented in accordance with the relevant regulations (Unique identifier: S2021-326-02). The working flow of this study was showed in Figure 2.

### 2.2. Baseline sociodemographic and clinical characteristics

Sociodemographic characteristics were directly collected from the medical records, including age, gender, height, weight, marital status, smoking history and alcohol use. The laboratory indices included triglycerides, total cholesterol, high-density lipoprotein cholesterol (HDL-C), low-density lipoprotein cholesterol (LDL-C), and blood glucose. Medical history was recorded, including hypertension, diabetes, hyperlipidemia and stroke, myocardial infarction, postural hypotension, medication history included antihypertensive and lipid lowering drugs, *etc*. Body mass index (BMI) was calculated as weight/height^2^ (kg/m^2^).

### 2.3. BP data collection

The study exposure was from after 1 January 2007 until the diagnosis of MCI, dementia, or the end of follow-up. All of the BP and pulse measurements were obtained in the morning on the right arm after at least 5 minutes of rest by the same instrument (Omron Healthcare, Inc., Kyoto, Japan) with the participant in a supine position. SBP, diastolic blood pressure (DBP) and pulse values measured during follow-up were averaged. PP was calculated as SBP-DBP, mean arterial pressure (MAP) was calculated as DBP + PP/3 and the PP index (PPI) was calculated as PP/SBP. Patients were grouped into 3 SBP (< 120, 120-135, ≥ 135 mm Hg) and DBP (<70, 70–80, ≥80 mm Hg) categories, and MAP, PP, PPI and pulse were categorized by their three tertiles. Those with SBP of 120–135 mm Hg, DBP of 70–80 mm Hg, or MAP, PP, PPI and pulse in the second tertile were considered the reference group.

### 2.4. Cerebral MRA acquisition and posterior circulatory artery stenosis analysis

All subjects underwent cerebral 3D-TOF MRA examinations with 3.0 T MR scanners of different brands, including a GE Signa HD (Milwaukee, WI, USA), GE 750 discovery (Milwaukee, WI, USA), and Siemens Skyra (Erlangen, Germany), with the following parameters: FOV 22 cm × 22 cm, slice thickness 1.4 mm, matrix 384 × 256. The repetition time (TR) and echo time (TE) were 9.7 msec/3.7 msec for the GE Signa HD, 34 ms/minimum for the GE 750 discovery and 21 ms/3.43 ms for the Siemens Skyra.

**FIGURE 3:**
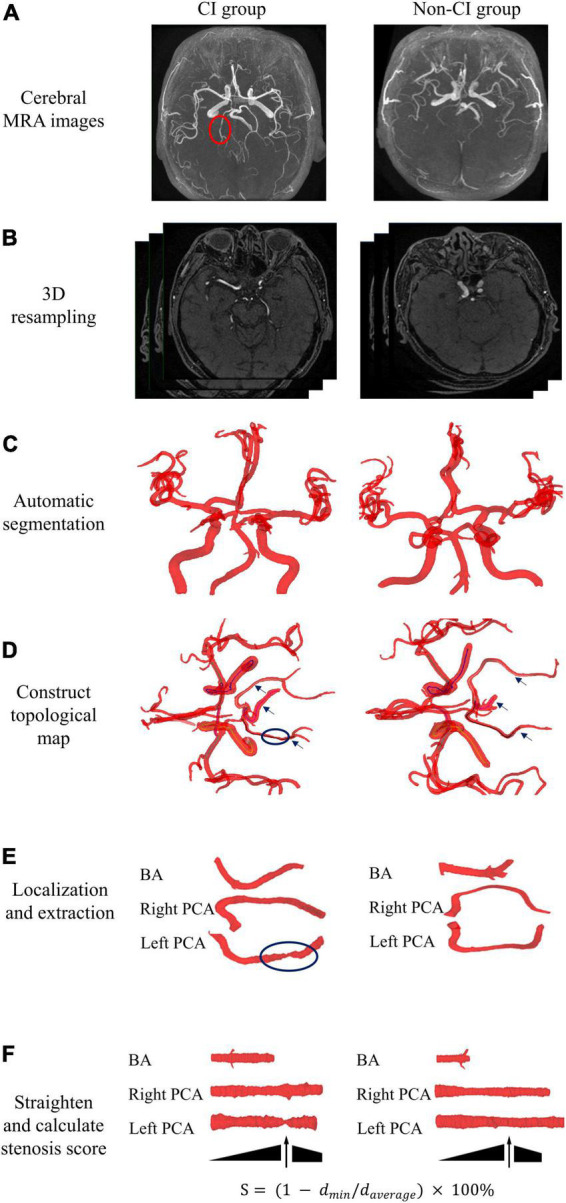
Overall workflow of automated quantitative analysis of posterior circulatory artery in cerebral MRA. Representative cerebral MRA images at CI and non-CI group. **(A)** Cerebral MRA data were collected. **(B)** 3D resampling of raw images was used to enhance axial resolution of 3D TOF MRA. **(C)** Automatic segmentation of cerebral arteries. **(D)** Construction of a topological map based on centerlines. **(E)** Automated localization and extraction of posterior circulatory artery. **(F)** Computational straightening and calculation of the stenosis score for target artery. BA, basilar artery; PCA, posterior cerebral artery; 3D TOF MRA, three dimensional time-of-flight magnetic resonance angiography.


The automated quantitative analysis method of geometry was applied to determine the maximum stenosis score of the posterior circulatory artery, including basilar artery (BA), and right and left posterior cerebral artery (PCA). Figure 3 shows the detailed procedure for the automated analysis of arterial maximum stenosis score in CI and non-CI group. Briefly, the image intensity thresholding and watershed algorithms were applied to segment the posterior circulatory arteries after 3D resampling of the raw images of 3D-TOF MRA. A skeletonization algorithm was used to extract the centerlines of segmented cerebral arteries and a topological map was constructed. Target artery was localized and extracted by shape features and complete centerline coordinates of individual arteries. Each extracted artery was computationally straightened through rotation and translation of their vascular cross-sections. The maximum stenosis score (S) of target cerebral artery was automatically calculated using the following equation.

(1) $$\text{S}=\left(1-d_{min}d_{average}\right)\times 100\%.......................................$$

where *d*_*min*_ denotes the minimum diameter of the artery analyzed, and *d*_*average*_ denotes the average diameter of the artery analyzed.

The stenosis grades of posterior circulatory artery were categorized as < 50% and ≥ 50% according this quantitative method. The accuracy of grade of posterior circulatory artery stenosis has been independently verified by two experienced radiologists.

### 2.5. Construction and evaluation of machine learning model

The means of various BP parameters, the posterior circulatory artery stenosis scores and other clinical information were used to construct predictive models for CI with four machine learning methods (logistic regression [LR], decision tree [DT], random forest [RF], and support vector machine [SVM])^1^ of 5-fold cross-validation. Receiver operating characteristic (ROC) curve analysis with the area under the curve (AUC) was used to study the ability of each model to predict the onset of CI. All individuals were randomly divided into the training cohort and validation cohort with a rate of 8:2.

## 3. Statistical analysis

All data were analyzed with SPSS (version 26.0, IBM) and R (version 4.1, see footnote 1). In per-patient analysis, continuous clinical variables at baseline between two groups were analyzed using the independent sample *t* test or Mann–Whitney U test, while categorical variables were analyzed using the Chi-squared (χ^2^) test. Case-control matching was used to select control group (1:1 matching) with the following variables: gender (±0) and age (±1). To investigate whether the association between the means of various BP parameters, posterior circulatory artery stenosis score, and clinical information and CI, LR analysis was conducted by continuations and categories of BP level and posterior circulatory artery stenosis score. These analyses were univariate and multivariate by adjusting for BMI, previous myocardial infarction history, and previous stroke history. In the correlations between BP level and posterior circulatory artery stenosis score of continuations, LR analyses were adjusted for BP parameters (SBP, DBP, and pulse) in model 1, BP parameters plus clinical indicators (BMI, previous myocardial infarction history, and previous stroke history) in model 2, and BP parameters, clinical indicators plus posterior circulatory artery stenosis score (BA stenosis score, right and left PCA stenosis scores) in model 3, respectively. Four machine learning methods (LR, DT, RF, and SVM) were used to construct multivariable predictive models for CI. The rpart package was used for DT analysis. The randomForest package was used for RF analysis. The e1071 package was used for RF analysis. The pROC package was used to plot ROC curves. The Delong test was used to test whether the difference between two ROC curves was statistically significant. A two-tailed p < 0.05 was regarded as statistically significant.

## 4. Results

### 4.1. Baseline clinical characteristics

A total of 208 patients of CI were recruited with BP data, and cerebral MRA was performed over the course of the study period (3.46 ± 3.19 years). After 1:1 case-control matching, a total of 416 subjects (age 84.47 ± 6.50 years old) were enrolled in this study. All subjects (89.4% male) received more than one BP measurement within the study period. The number of BP measurements per person was 56.98 ± 77.94, with the maximum being 576 measurements. The characteristics of the subjects are presented in Table 1. SBP, PP, and PPI were significantly higher in the CI group than in the control group (all *p* < 0.05). CI group patients were more likely to have previous diabetes, myocardial infarction, and stroke (all *p* < 0.05). In addition, 103 patients with BP and high-quality cerebral MRA were selected as the CI group, with a time from MRA to CI diagnosis of 4.32 ± 3.22 years, and 100 subjects without CI were enrolled as the control group (Supplementary Table 1). The stenosis score of the left PCA was significantly higher in the CI group than in the control group (*p* < 0.05) (Table 1).

**TABLE 1:** Baseline characteristics of patients enrolled in this study.

Baseline characteristics	Overall	Non-CI group (*n* = 208)	CI group (*n* = 208)	*P*-value
**Demographics**				
Age (years)	85 (81–89)	85 (80–88)	86 (81–89)	0.249
Male, *n* (%)	372 (89.4)	186 (89.4)	186 (89.4)	–
Body mass index (kg/m^2^), mean ± SD	24.32 ± 3.16	25.58 ± 3.12	24.05 ± 3.18	0.085
Married/widowed, *n* (%)	334 (80.3)	166 (79.8)	168 (80.8)	0.805
Years of schooling (years)	12 (10,12)	12 (10,12)	12 (12,12)	0.081
Smoking, *n* (%)	138 (33.2)	71 (34.1)	67 (32.2)	0.677
Drinking, *n* (%)	80 (19.2)	41 (19.7)	39 (18.8)	0.804
**Blood pressure profile**				
SBP (mmHg)	132.00 (125.50–137.74)	130.03 (124.00–136.00)	132.96 (126.70–138.96)	0.005
DBP (mmHg)	70.00 (65.71–73.65)	70.19 (65.74–73.91)	69.32 (65.66–73.10)	0.168
PP (mmHg)	61.95 (56.40–67.70)	60.33 (54.59–66.80)	63.32 (57.80–69.38)	<0.001
MAP (mmHg)	90.20 (86.19–94.26)	90.33 (86.00–93.83)	90.03 (86.70–94.69)	0.456
PPI	0.47 (0.44–0.50)	0.46 (0.43–0.49)	0.48 (0.45–0.50)	0.001
Pulse (times/min)	70.13 (66.78–72.76)	70.33 (67.00–72.77)	70.01 (66.66–72.77)	0.538
**Cerebral artery stenosis score**				
BA (%)	15.26 (14.92–20.57)	15.25 (14.93–21.49)	15.26 (14.92–20.00)	0.865
Right PCA (%)	27.19 (15.47–46.22)	20.08 (15.22–40.00)	30.00 (16.48–51.00)	0.077
Left PCA (%)	25.07 (16.25–44.84)	20.16 (15.00–35.61)	29.45 (20.00–52.00)	0.001
**Medical history**				
Diabetes, *n* (%)	126 (30.3)	51 (24.5)	75 (36.1)	0.010
Hypertension, *n* (%)	300 (72.1)	153 (73.6)	147 (70.7)	0.512
Myocardial infarction, *n* (%)	23 (5.5)	6 (2.9)	17 (8.2)	0.018
Postural hypotension, *n* (%)	14 (3.4)	4 (1.9)	10 (4.8)	0.103
Hyperlipidaemia, *n* (%)	81 (19.5)	47 (22.6)	34 (16.3)	0.107
Stroke, *n* (%)	96 (23.1)	30 (14.4)	66 (31.7)	<0.001
**Clinical features**				
Triglycerides (mmol/L)	1.24 (0.92–1.70)	1.29 (0.99–1.73)	1.22 (0.90–1.70)	0.123
Total cholesterol (mmol/L)	4.19 (3.74–4.85)	4.19 (3.73–4.92)	4.25 (3.74–4.80)	0.895
HDL-C (mmol/L)	1.18 (1.02–1.45)	1.18 (1.00–1.44)	1.24 (1.03–1.45)	0.462
LDL-C (mmol/L)	2.55 (2.04–3.12)	2.63 (2.00–3.19)	2.53 (2.05–2.98)	0.360
Blood glucose (mmol/L)	5.39 (4.90–6.03)	5.46 (4.95–6.03)	5.36 (4.86–6.01)	0.322
**Medication use**				
Antihypertensive drugs, *n* (%)	265 (63.7)	140 (67.3)	125 (60.1)	0.126
Lipid-lowering drugs, *n* (%)	224 (53.8)	118 (56.7)	106 (51.0)	0.238

Normal distribution values are expressed as mean ± standard error of mean (SEM); abnormal distribution values are expressed as median (Q1–Q3). SBP, systolic blood pressure; DBP, diastolic blood pressure; PP, pulse pressure; MAP, mean arterial pressure; PPI, pulse pressure index; BA, basilar artery; PCA, posterior cerebral artery; HDL-C, high-density lipoprotein cholesterol; LDL-C, low-density lipoprotein cholesterol.

### 4.2. LR analysis of the relationship between the means of various BP parameters, posterior circulatory artery stenosis score and the risk of CI

The association between the means of various BP parameters (SBP, PP, and PPI) and CI remained significant (all *p* < 0.05) (Table 2). Increased SBP (odds ratio [OR] 1.027 [per mm Hg]), 95% confidence interval (CI): 1.008–1.047) and PP (OR 1.040 [per mm Hg], 95% CI: 1.017–1.063) increased the risk of CI after adjustment for BMI, previous myocardial infarction history, and previous stroke history. PP in the first tertile (<58 mm Hg) (OR 0.588, 95% CI: 0.362–0.955) decreased the risk of CI. It is worth noting that increased PPI (OR 2077.952, 95% CI: 20.712–208,473.642) markedly increased the risk of CI. The stenosis score of the left PCA (OR 1.021 [per cent], 95% CI: 1.005–1.038) and stenosis ≥ 50% of the left PCA (OR 2.854, 95% CI: 1.387–5.872) increased the risk of CI.

**TABLE 2:** Logistic regression analysis for the relationship between risk factors and CI.

Variables	Model a OR (95% CI)	Model b OR (95% CI)
**BP parameters**		
SBP (mm Hg)	1.029 (1.010–1.049)*	1.027 (1.008–1.047)*
Stage I (<120)	0.874 (0.456–1.674)	0.854 (0.437–1.667)
Stage II (120–135)	–	–
Stage III (≥135)	1.571 (1.030–2.396)*	1.514 (0.979–2.340)
DBP (mm Hg)	0.984 (0.953–1.016)	0.988 (0.955–1.022)
Stage I (<70)	1.461 (0.982–2.175)	1.357 (0.892–2.065)
Stage II (70–80)	–	–
Stage III (≥80)	1.338 (0.562–3.185)	1.273 (0.518–3.125)
MAP (mm Hg)	1.017 (0.987–1.048)	1.018 (0.987–1.050)
First tertile (<88)	0.769 (0.478–1.237)	0.709 (0.432–1.163)
Second tertile (88–93)	–	–
Third tertile (≥93)	0.911 (0.567–1.464)	0.845 (0.516–1.383)
PP (mm Hg)	1.043 (1.021–1.065)*	1.040 (1.017–1.063)*
First tertile (<58)	0.584 (0.364–0.934)	0.588 (0.362–0.955)*
Second tertile (58–66)	–	–
Third tertile (≥66)	1.042 (0.652–1.666)	1.006 (0.620–1.633)
PPI	3588.920 (44.392–290148.336)*	2077.952 (20.712–208473.642)*
First tertile (<0.45)	0.625 (0.390–1.002)	0.621 (0.381–1.013)
Second tertile (0.45–0.49)	–	–
Third tertile (≥0.49)	1.529 (0.955–2.449)	1.522 (0.934–2.481)
Pulse (times/min)	0.987 (0.949–1.027)	0.984 (0.944–1.024)
First tertile (<68)	1.409 (0.882–2.253)	1.335 (0.821–2.171)
Second tertile (68–72)	–	–
Third tertile (≥72)	1.128 (0.706–1.802)	1.034 (0.635–1.684)
**Cerebral artery stenosis score**		
BA (%)	1.000 (0.973–1.028)	0.998 (0.970–1.026)
<50% stenosis	–	–
≥50% stenosis	1.224 (0.319–4.698)	1.224 (0.311–4.824)
Right PCA (%)	1.013 (0.999–1.027)	1.011 (0.997–1.026)
<50% stenosis	–	–
≥50% stenosis	1.568 (0.817–3.007)	1.493 (0.766–2.910)
Left PCA (%)	1.022 (1.006–1.038)*	1.021 (1.005–1.038)*
<50% stenosis	–	–
≥50% stenosis	2.769 (1.372–5.588)*	2.854 (1.387–5.872)*

SBP and DBP were formed into three stages. PP, MAP, and PPI were categorized into three stages according to the tertiles. Model a: risk factor of CI; Model b: model a + BMI, previous myocardial infarction history, and previous stroke history. SBP, systolic blood pressure; DBP, diastolic blood pressure; PP, pulse pressure; MAP, mean arterial pressure; PPI, pulse pressure index; BA, basilar artery; PCA, posterior cerebral artery; BMI, body mass index.

*Represents *p* < 0.05.

**FIGURE 4:**
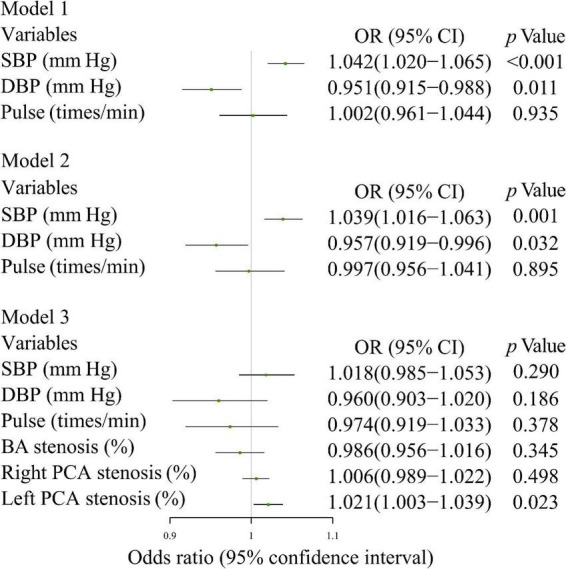
Multivariate logistic regression analysis of the relationship between the means of various BP parameters and CI. Model 1: SBP, DBP, and Pulse; Model 2: model 1 + BMI, previous myocardial infarction history, and previous stroke history; Model 3: model 1 + BA stenosis score, right and left PCA stenosis scores. SBP, systolic blood pressure; DBP, diastolic blood pressure; PP, pulse pressure; MAP, mean arterial pressure; PPI, pulse pressure index; BMI, body mass index; BA, basilar artery; PCA, posterior cerebral artery.

Increased SBP (OR 1.042 [per mm Hg], 95% CI: 1.020-1.065) increased the risk of CI after adjustment for DBP and pulse; it also increased the risk of CI (OR 1.039 [per mm Hg], 95% CI: 1.016-1.063) after adjustment for DBP, pulse, BMI, previous myocardial infarction, and stroke history. Increased stenosis score of the left PCA (OR 1.021 [per cent], 95% CI: 1.003-1.039) was associated with an increased the risk of CI after adjustment for SBP, DBP, pulse, stenosis scores of BA and right PCA (Figure 4).

### 4.3. The correlations between the means of various BP parameters and posterior circulatory artery stenosis scores

**FIGURE 5:**
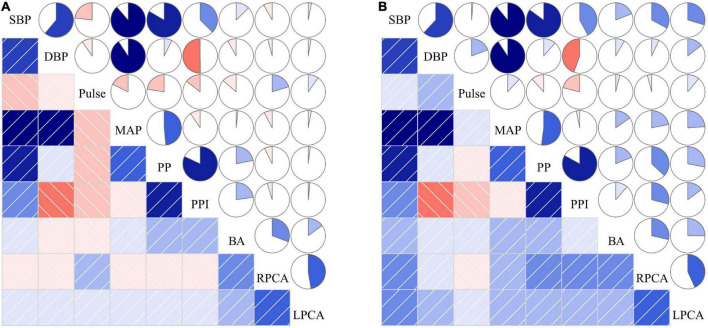
The correlations between the means of various BP parameters and posterior circulatory artery stenosis score in CI **(A)** and non-CI **(B)** group. The correlations among risk factors can be categorized into two areas in the figure, the lower left and the upper right area. The lower left area: The blue squares with slashes starting from lower left corner to upper right corner indicate positive correlation of the two variables. The red squares with slashes starting from upper left corner to lower right corner indicate negative correlation of the two variables. The darker the color, the stronger the correlation of the two variables. The upper right area: The pie charts in blue which are filled in a clockwise direction are expressed as positive correlations of the two variables. The pie charts in red which are filled in a counterclockwise direction are expressed as negative correlations of the two variables. The larger the marked color area in the pie chart, stronger the correlation between the two variables. SBP, systolic blood pressure; DBP, diastolic blood pressure; MAP, mean arterial pressure; PP, pulse pressure; PPI, pulse pressure index; BA, the stenosis score of basilar artery; RPCA, the stenosis score of right posterior cerebral artery; LPCA, the stenosis score of left posterior cerebral artery.

Figure 5 showed no significant correlation between the means of various BP parameters and posterior circulatory artery stenosis scores. Although increased SBP, DBP, MAP, PP, and PPI were positively associated with the stenosis scores of BA, right and left PCA in non-CI group, their related coefficients were also less than 0.5. The correlations between the means of various BP parameters and posterior circulatory artery stenosis grade in CI and non-CI group was shown in Supplementary Figure 1. Increased SBP, DBP, MAP, PP, and PPI were also positively associated with the stenosis grades of BA, right and left PCA in non-CI group.

### 4.4. Prediction model for the risk of CI

**FIGURE 6:**
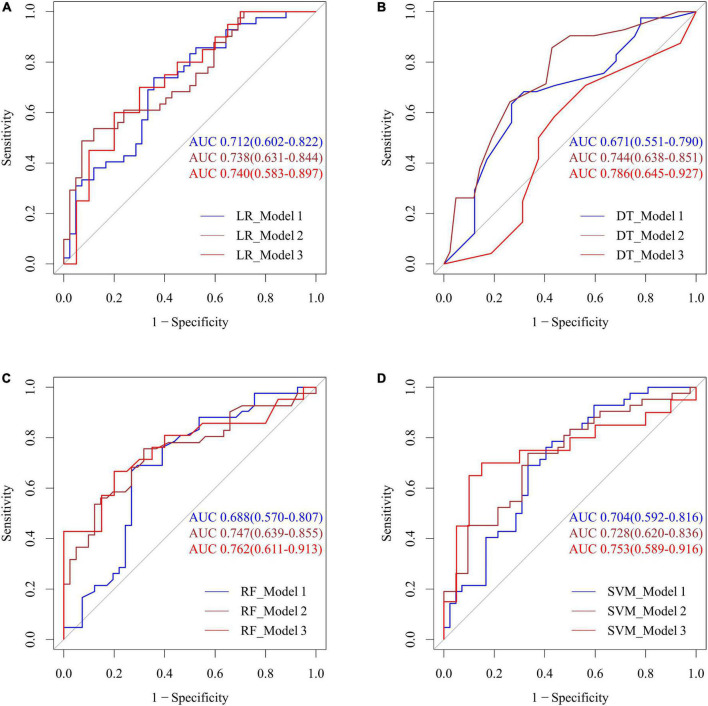
Performance of four machine learning methods including LR **(A)**, DT **(B)**, RF **(C)**, and SVM **(D)** with the means of various BP parameters, posterior circulatory artery stenosis score for predicting CI. Model 1: SBP, DBP, pulse; Model 2: model 1 + BMI, previous myocardial infarction history, and previous stroke history; Model 3: model 2 + BA stenosis score, right and left PCA stenosis scores. SBP, systolic blood pressure; DBP, diastolic blood pressure; BMI, body mass index; LR, logistic regression; DT, decision tree; RF, random forest; SVM, support vector machine.

The predictive ability of the different models incorporating the means of the various BP parameters (SBP, DBP, and pulse), clinical indicators and posterior circulatory artery stenosis scores was shown in Figure 6. Combining SBP, DBP, and pulse, the LR, DT, RF, and SVM models predicted CI with AUCs of 0.712, 0.671, 0.688, and 0.704, respectively. To improve the efficiency of the above model, we further integrated the posterior circulatory artery stenosis scores, clinical indicators into the prediction model to form another model, with AUCs of 0.740, 0.786, 0.762, and 0.753, respectively (all *p* > 0.05).

## 5. Discussion

In this population-based retrospective nested case-control cohort study of elderly Chinese adults, we found that PP in the first tertile (< 58 mmHg) was negatively associated with CI progression, while ≥ 50% stenosis of the left PCA was positively associated with CI progression. The hybrid model combined with the stenosis score of posterior circulatory artery and the means of various BP parameters can effectively predict the risk of CI in elderly individuals.

Hypertension, one of the most common and potentially modifiable dementia risk factors, has the highest population attributable fraction (5%) among all vascular risk factors (Livingston et al., 2017). The interaction between hypertension and aging is multifaceted and particularly increases the risk of vascular CI and Alzheimer’s disease (Iadecola et al., 2016; Cortes-Canteli and Iadecola, 2020; Ungvari et al., 2021). We found that elevated SBP was associated with an increased risk of CI in the present study. Elevated SBP was associated with poorer cognitive performance, particularly delayed recall (Hestad et al., 2021), which is consistent with the results of our study. A meta-analysis indicated that midlife SBP > 130 mm Hg was related to an increased dementia risk (Ou et al., 2020). Although we found that elevated DBP was related to a reduction in CI risk after adjusting for SBP and pulse, the results should be carefully interpreted. The first reason is that the value of the OR is close to one, and may not appear to be clinically significant. Another reason is that there was no statistical significance in the relationship between elevated DBP and CI on univariate LR analysis.

High PP may induce instability of carotid plaque and cause cerebral infarction (Crespo-Cuevas et al., 2020). Hypertension-induced pathology can accelerate vascular aging and enhance the progression of atherosclerotic plaques in the main cerebral arteries, which may decrease cerebral blood flow and cause cognitive deficits in elderly individuals (Iadecola and Gottesman, 2019; Ungvari et al., 2021). When PP levels were greater than 50 or 55 mm Hg, higher PP was related to cardiovascular events (Ren et al., 2021), consistent with our results. Our results showed that a PP less than 58 mm Hg was considered to reduce the risk of MCI. Lower PP levels are thought to decrease the risk because elderly adults are more prone to atherosclerosis and stroke. Excessive fluctuation of arterial-pressure and damaged hippocampal capillaries may cause large arteries to become stiffer and less compliant, especially among the aging population (De Montgolfier et al., 2019; Ren et al., 2021). Vascular alterations may contribute to reduced perfusion of the cerebral white matter and ultimately increase the risk of CI (Iadecola, 2017).

The posterior circulatory artery stenosis scores, extracted by the automated quantitative analysis method, was assessed for its relationship with BP and CI in this study. The posterior circulation artery stenosis score was used as a continuous variable to assess the risk of dementia, which was one of the highlights of this study. We found that there was a positive correlation between the left PCA stenosis score and CI risk, and ≥ 50% stenosis of the left PCA was associated with an increased risk for CI. Intracranial arterial stenosis is an independent risk factor for dementia, MCI, and vascular CI (Hilal et al., 2017; Jennifer et al., 2017a; Suri et al., 2018; Crespo-Cuevas et al., 2020). Stenosis of the PCA may decrease cerebral blood flow to the posterior cingulate and hippocampus, leading to impairment of cognitive functioning, executive functioning and memory processing (Liu et al., 2018; Watanabe et al., 2019; Li et al., 2020; Maarten et al., 2020). Infarcts of the left frontotemporal lobes and left thalamus are strongly associated with poststroke CI. The left frontotemporal lobes and thalamus are supplied by the left PCA, and narrowing causes inadequate perfusion or microinfarction, leading to a decline in cognitive function (Yang et al., 2022). In this study, there was no significant correlation between BP and the posterior circulatory artery stenosis scores by Spearman correlation analysis. A cross-sectional study recruited 3640 participants and found similar association between DBP and intracranial artery stenosis with us (Song et al., 2020). The DBP in this elderly population was relatively lower than that in the previous study and the predictive value of DBP decreased with the aging (Wang et al., 2015).

Machine learning was used to obtain the highest level features automatically from existing data for classification (Fu et al., 2020). Although OR and hazard ratio values were used in most studies to represent the relationship between BP and CI (Ernst et al., 2021; Hestad et al., 2021), we focused on the AUC of predictive model in this study. To improve the efficiency of the models in the prediction of CI risk, we further integrated the posterior circulation artery stenosis score and clinical indicators into the prediction model, together with the means of various BP parameters included in original LR, DT, RF and SVM models. The underlying reason may be that abnormal BP and posterior circulation artery stenosis were most important vascular risk factors (Jennifer et al., 2017b; Livingston et al., 2017). In addition, machine learning is regarded as a reliable method to construct and improve predictive models with available data, so as to contribute to decisions on clinical diagnosis and treatment (Ding et al., 2019; Moscoso et al., 2019). Since identifying the high-risk population for CI is essential, it is of great significance to enhance individualized prevention strategies for CI of BP and artery stenosis management (Lv et al., 2017). Based on this, MCI or dementia could be screened at earlier stage (Lane et al., 2019). Additionally, attaching great importance to CI and developing neurodegenerative encephalopathy markers (including biological, genetic and cognitive markers) to identify the similarities and differences between dementia subtypes and to understand the progression from early to late stages (Veleva et al., 2021; Yan et al., 2021; Zetterberg and Bendlin, 2021). A prevention strategy should be developed to thwart CI and slow down disease progression by targeting the identified risk factors (Dallaire-Theroux et al., 2021).

There were some limitations in this study. The participants were recruited from a single medical center, which may not necessarily be representative of all Chinese individuals. Second, the sample size is relatively small, causing machine learning overfitting and reducing the performance of the models. Because a proportion of the elderly individuals had atrial fibrillation, the pulse may not reflect the resting heart rate in this study. Most of the patients achieved ideal hypertension control that might affect the influences of BP on CI in this study. Finally, the multivariate predictive models were descriptive and only reflected the ability to predict MCI or dementia in this study. High-quality large-scale clinical trials with large samples, external validation, and long-term follow-up with prospective cohorts are required to further validate our findings.

## 6. Conclusion

Elderly with low pulse differential pressure may have lower risk for cognitive impairment. Combining the stenosis scores of posterior circulatory arteries with the means of various BP parameters may effectively predict the risk of CI. Further multicentre prospective studies are still needed to determine the comprehensive parameters of biomarkers and radiomics that can identify the underlining mechanisms of progress between the subtypes of dementia.

## Data availability statement

The original contributions presented in this study are included in the article/Supplementary material, further inquiries can be directed to the corresponding authors.

## Ethics statement

The studies involving human participants were reviewed and approved by Chinese PLA General Hospital. Written informed consent for participation was not required for this study in accordance with the national legislation and the institutional requirements.

## Author contributions

FC and LF conceived and designed the experiments, provided financial support, and co-wrote and edited the manuscript. SS, L-BC, and XX performed the experiments. GY, YZ, and YG calculated cerebral arterial stenosis scores. DL, TS, and JJ collected and analyzed the data. NL, YW, SL, and XW provided technical support. All authors contributed to the article and approved the submitted version.

## Footnotes

1
https://www.r-project.org/RStudio

## Supplementary material

Supplementary Figure 1 The correlations between the means of various BP parameters and posterior circulatory artery stenosis grade in CI **(A)** and non-CI **(B)** group.
