# Supplementary figures and images for: Longitudinal real world correlation study of blood pressure and novel features of cerebral magnetic resonance angiography by artificial intelligence analysis on elderly cognitive impairment
Source: Front Aging Neurosci. 2023 Feb 3;15:1121152. doi: 10.3389/fnagi.2023.1121152 (PMC9935573; doi:10.3389/fnagi.2023.1121152)

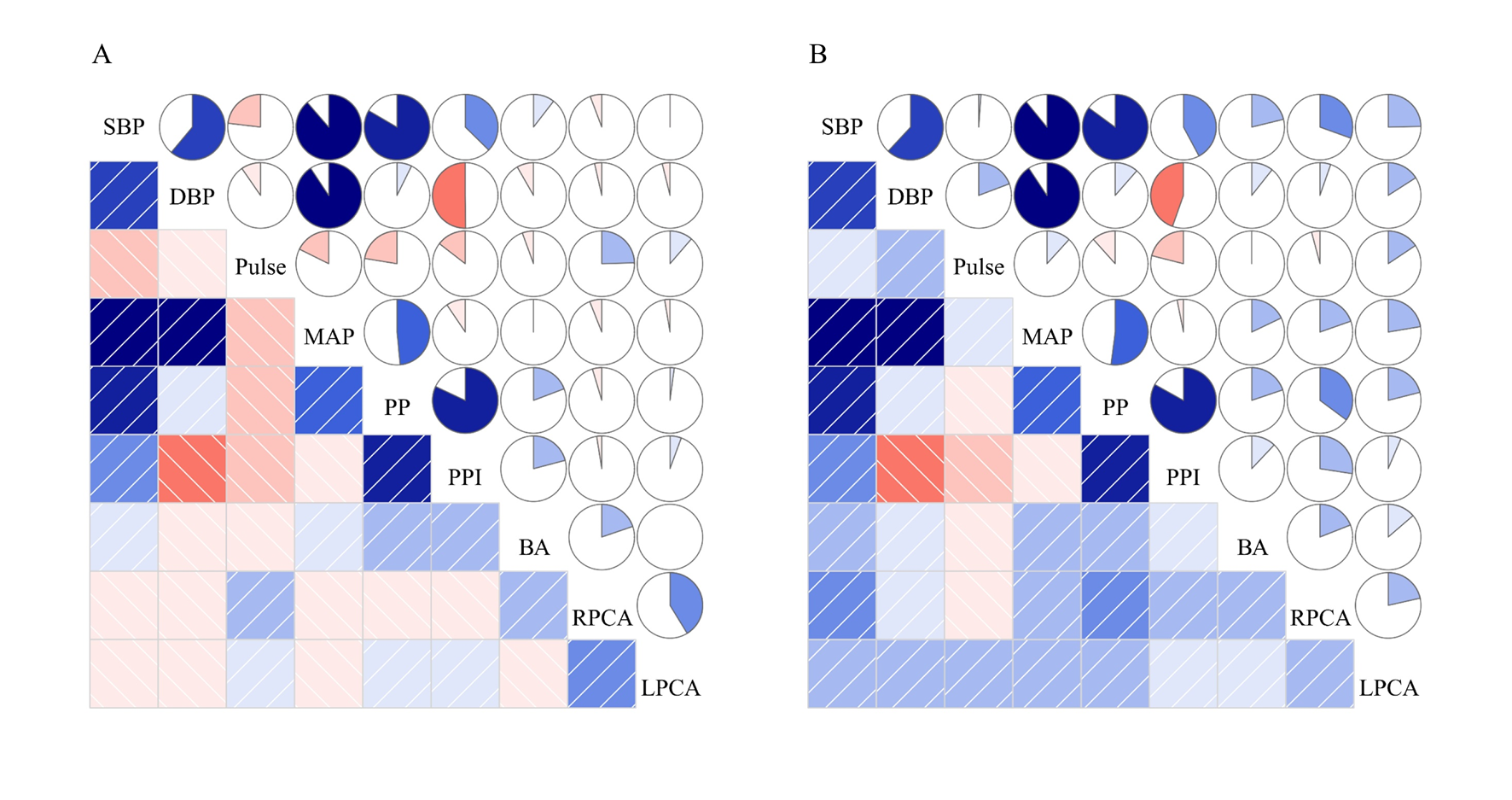

Supplement: Supplementary Figure 1 — The correlations between the means of various BP parameters and posterior circulatory artery stenosis grade in CI (A) and non-CI (B) group. [file Image_1.tif]
