# Supplementary material for: Longitudinal real world correlation study of blood pressure and novel features of cerebral magnetic resonance angiography by artificial intelligence analysis on elderly cognitive impairment
Source: Front Aging Neurosci. 2023 Feb 3;15:1121152. doi: 10.3389/fnagi.2023.1121152 (PMC9935573; doi:10.3389/fnagi.2023.1121152)
Supplement: Supplementary file 2 [file Table_1.docx]

**Supplementary TABLE 1.**  **Characteristics of patients with BP and cerebral MRA**

| Characteristics | Non-CI group (n=100) | CI group (n=103) | *p*-value |
| --- | --- | --- | --- |
| Demographics |  |  |  |
| Age (years) | 85(80-88) | 85(79-88) | 0.249 |
| Male, n (%) | 88(88.0) | 90(87.4) | 0.893 |
| Body mass index (kg/m^2^), mean ± SD | 23.94±3.43 | 23.98±3.39 | 0.939 |
| Married/widowed, n (%) | 100(100.0) | 103(100.0) | - |
| Years of schooling (years) | 10(10,12) | 10(10,12) | 0.614 |
| Smoking, n (%) | 38(38.0) | 33(32.0) | 0.373 |
| Drinking, n (%) | 16(16.0) | 19(18.4) | 0.645 |
| Blood pressure profile |  |  |  |
| SBP (mmHg) | 129.95(123.90- 137.69) | 133.00(126.50-138.27) | 0.303 |
| DBP (mmHg) | 69.29(66.51-73.55) | 68.83(65.27- 73.02) | 0.445 |
| PP (mmHg) | 60.83(56.17-67.84) | 62.81(58.25-68.24) | 0.126 |
| MAP (mmHg) | 89.35(85.82-93.58) | 89.19(85.87-94.59) | 0.934 |
| PPI | 0.47(0.44-0.49) | 0.48(0.46-0.50) | 0.063 |
| Pulse (times/min) | 70.27(67.23-73.08) | 69.37(66.45-72.28) | 0.442 |
| Medical history |  |  |  |
| Diabetes, n (%) | 22(22.0) | 31(30.1) | 0.189 |
| Hypertension, n (%) | 65(65.0) | 68(66.0) | 0.879 |
| Myocardial infarction, n (%) | 5(5.0) | 7(6.8) | 0.587 |
| Postural hypotension, n (%) | 1(1.0) | 1(1.0) | 0.983 |
| Hyperlipidaemia, n (%) | 16(16.0) | 12(11.7) | 0.369 |
| Stroke, n (%) | 13(13.0) | 29(28.2) | 0.008 |
| Clinical features |  |  |  |
| Triglycerides (mmol/L) | 1.22(0.90-1.64) | 1.21(0.88-1.70) | 0.923 |
| Total Cholesterol (mmol/L) | 4.08(3.52-4.77) | 4.26(3.80-4.83) | 0.231 |
| HDL-C (mmol/L) | 1.21(0.98-1.55) | 1.25(1.01-1.46) | 0.536 |
| LDL-C (mmol/L) | 2.43(1.88-3.06) | 2.55(2.09-2.99) | 0.481 |
| Blood glucose (mmol/L) | 5.35(5.01-5.89) | 5.27(4.88-6.01) | 0.727 |
| Medication use |  |  |  |
| Antihypertensive drugs, n (%) | 50(50.0) | 54(52.4) | 0.729 |
| Lipid-lowering drugs, n (%) | 46(46.0) | 48(46.6) | 0.931 |

Normal distribution values are expressed as mean ± standard error of mean (SEM); abnormal distribution values are expressed as median (Q1–Q3).

SBP: systolic blood pressure; DBP: diastolic blood pressure; PP: pulse pressure; MAP: mean arterial pressure; PPI: pulse pressure index; HDL-C: high-density lipoprotein cholesterol; LDL-C: low-density lipoprotein cholesterol.
